# Aortic Dissection Presenting as Acute Subtotal Left Main Coronary Artery Occlusion: A Case Approach and Review of the Literature

**DOI:** 10.14740/jocmr2039w

**Published:** 2015-03-01

**Authors:** Michael Ruisi, Arzhang Fallahi, Moinakhtar Lala, Yumiko Kanei

**Affiliations:** a Department of Cardiology, Mount Sinai Beth Israel, New York, NY 10003, USA

**Keywords:** Aortic dissection, Left main coronary artery, Occlusion

## Abstract

Aortic dissection is the most common fatal condition of the aorta, yet it is often missed on initial clinical presentation. Aortic dissection associated with acute coronary syndrome (ACS) is relatively rare, but if it occurs, it can be diagnostically challenging, and the condition can be fatal. Here we describe a case of aortic dissection presenting as ST-segment elevation myocardial infarction (STEMI) managed via the transradial approach. We describe the current literature on the subject.

## Introduction

Aortic dissection is a medical and surgical emergency that occurs in two out of every 10,000 people between 40 and 70 years of age. Typical patient presentation involves the symptoms of chest pain with radiation to the back and scapular regions. On clinical exam, patients may have discrepancies in blood pressure between right and left arms as well as auscultation of a diastolic murmur. CT angiography with dissection protocol has been the diagnostic imaging modality used with highest yield in the acute setting. However, other simpler diagnostic measures such as ECG analysis and chest X-ray can offer clues to the diagnosis. In the setting where CT angiography cannot be utilized, trans-esophageal echocardiography offers diagnostic assessment. Clinical presentation spans the spectrum of significant discomfort with normal vitals, to cardiogenic shock, and sudden death [1-3]. Aortic dissection can mimic other cardiovascular emergencies providing challenging diagnostic dilemmas for physicians. Unfortunately, timely recognition of this syndrome has significant and direct impacts on patient mortality. We present the case of a 61-year-old male with acute onset of substernal chest pain with initial ECG analysis consistent with ST elevation myocardial infarction found to have a type A aortic dissection on coronary angiography.

## Case Report

**Figure 1:**
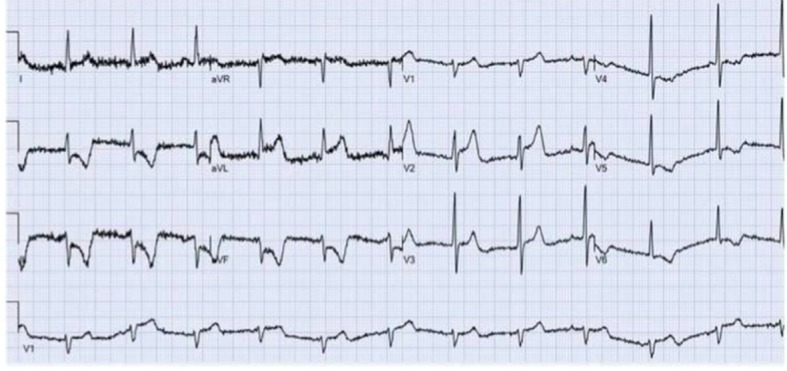
Presenting 12-lead EKG revealing ST elevations in leads I, AVL, V1-V3 with significant ST depressions in the inferior leads.

A 61-year-old male with a past medical history of hypertension presented to our emergency room with the acute onset of substernal chest pain that began 45 min prior to arrival to the emergency department. The pain was characterized as pressure-like, progressive in nature with radiation to the back and interscapular region. The presenting vital signs showed a blood pressure of 93/49 mm Hg, heart rate of 70 bpm, with normal oxygen saturation. An initial 12-lead ECG revealed ST elevations in leads I, AVL, V1-V3 with significant ST depressions in the inferior leads (Fig. 1). The patient was given aspirin 325 mg, clopidogrel 600 mg, and a heparin bolus of 5,000 units. The patient was immediately taken to the catheterization lab for diagnostic angiogram and possible percutaneous coronary intervention.

**Figure 2:**
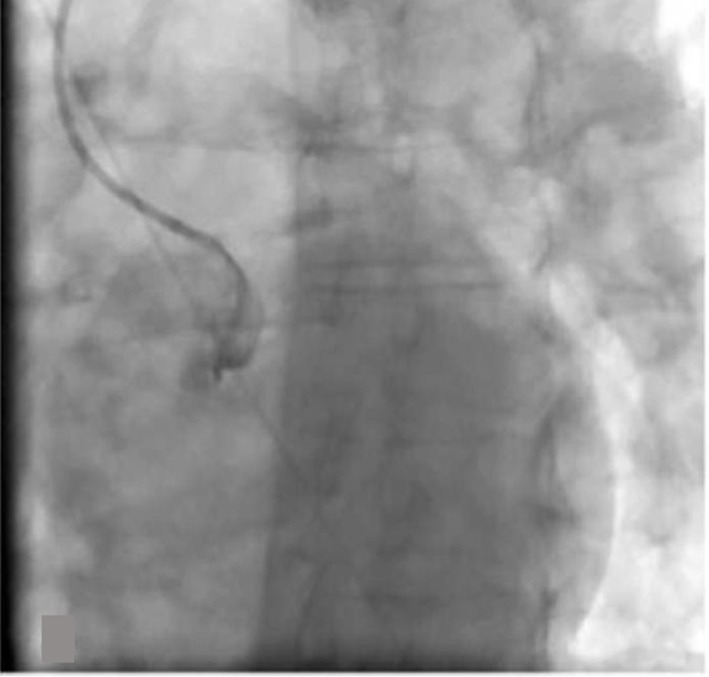
Subtotal occlusion of the left main coronary artery.

**Figure 3:**
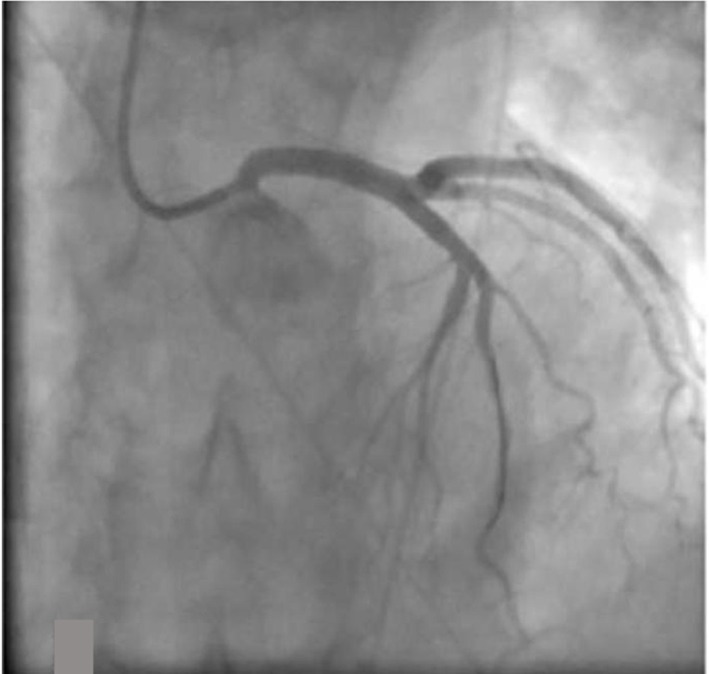
Post-stenting of left main artery with good angiographic result.

In the cath lab the right radial artery was cannulated with a 6-French sheath. A Launcher EBU 3.5 guide catheter (Medtronic, Minneapolis, MN) was used to engage the left coronary ostium. Coronary angiogram revealed a subtotal occlusion of the left main coronary artery (Fig. 2). A Runthrough wire (Terumo Medical Corporation, Somerset, NJ) was utilized to cross the lesion and a 3.0 x 15 mm Emerge balloon (Boston Scientific, Natick, MA) was used for initial angioplasty. Since patient became hypotensive, intraaortic balloon pump was placed from the right femoral artery. Two 4.0 × 12 mm Promus Element Everolimus-Eluting stents (Boston Scientific, Natick, MA) were deployed in the left main artery with good angiographic result (Fig. 3). Intravascular ultrasound (IVUS) was performed to confirm the stent expansion.

**Figure 4:**
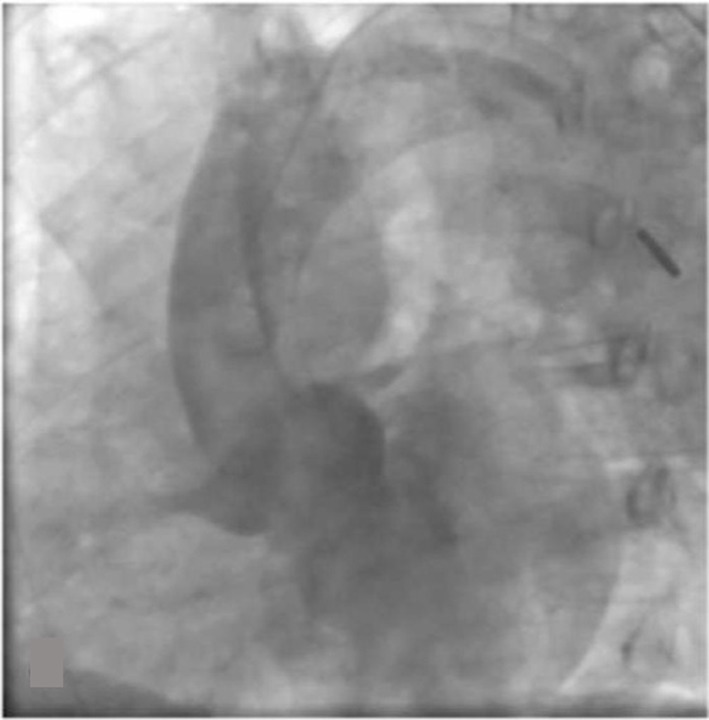
Aortogram revealing severe aortic regurgitation with possible dissection flap.

**Figure 5:**
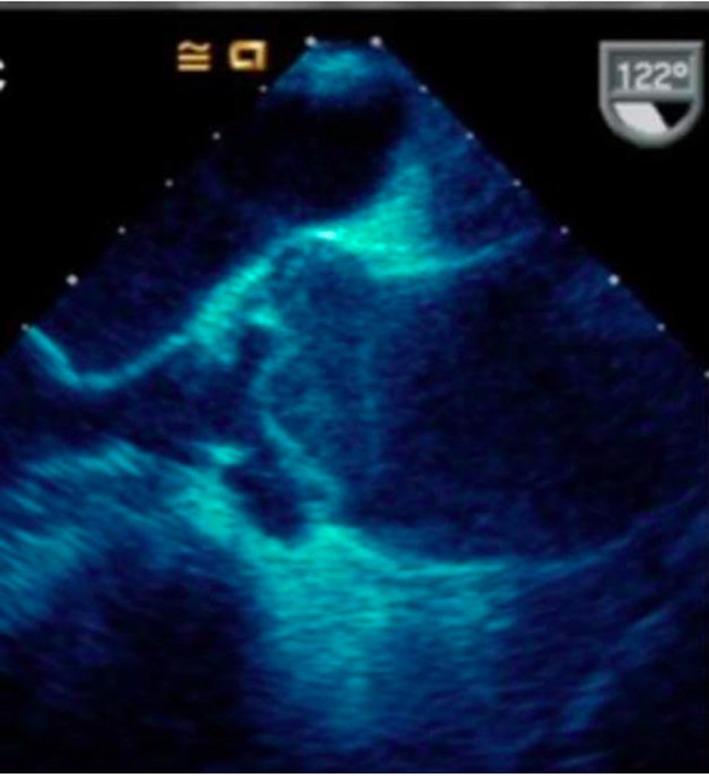
Trans-esophageal echocardiography confirming aortic dissection of the ascending aorta.

**Figure 6:**
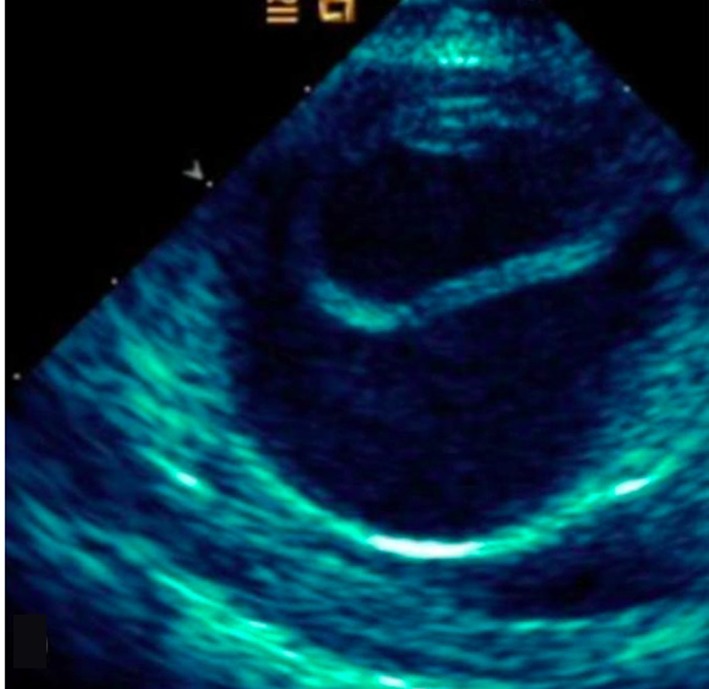
Short axis of aorta on TEE demonstrating dissection flap in lumen.

**Figure 7:**
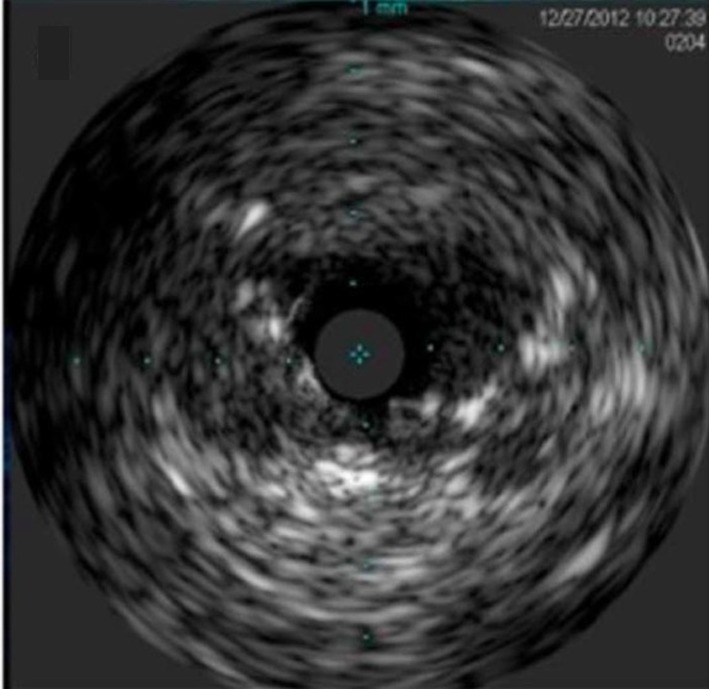
Intra-vascular ultrasound (IVUS) dynamic flap outside the left main ostium.

After the stent placement, the chest pain improved and the ST segments elevations resolved. However, the patient later continued to have persistent chest pain. On review of the angiogram, a significant amount of aortic insufficiency was noted. An aortogram was performed revealing severe aortic regurgitation with a possible dissection flap (Fig. 4). Urgent trans-esophageal echocardiography was performed in the cath lab confirming aortic dissection of the ascending aorta (Fig. 5, 6). On review of IVUS, dynamic flat was seen outside the left main ostium (Fig. 7). The patient underwent emergent repair of a type A aortic dissection.

In the operating room, patient developed PEA arrest, which required resuscitation, and the pericardium was opened revealing hemopericardium. The proximal aorta was then mobilized and the aorta resected to just above the sinotubular ridge. The three leaflets of the aortic valve appeared normal, and the dissection flap was noted to extend down into the non-coronary sinus. The left main coronary artery was intact but surrounded by a substantial hematoma external to the aorta. There was no evidence of extension of the dissection into the left main coronary artery. Post-op course was complicated by minor stroke, but patient was transferred to rehabilitation floor with good recovery.

## Discussion

One of the first reported cases of dissection presenting as ACS was in a patient with anterior ST elevations who was found to have an aortic intimal flap occluding the left coronary artery, resulting in cardiac arrest and death [2]. Myocardial ischemia has been reported in 19.9% of cases of aortic dissection and new Q wave or ST elevations in 6.0% of cases [3]. ST-segment elevation myocardial infarction (STEMI) during type A dissection is rare, but if it happens, it is a serious condition with high mortality rate if not recognized and treated promptly. The incidence of malperfusion of coronary artery in the patients who underwent surgery for type A aortic dissection is reported to be 6.1-11.3% [4, 5]. The initial presentation of STEMI was seen in 4.6% of patients, and involvement of right coronary artery (RCA) is more common than left main artery (eight inferior and two anterior in one series) [4].

Neri et al [5] classified the mechanism of coronary ostia involvement into three types. Type A is an ostial dissection, with a disruption of the inner layer limited to the area of the coronary ostium. The type B dissection, with a coronary false channel, is a retrograde extension of the aortic dissection extending into the coronary artery wall. Type C is circumferential detachment with an inner cylinder intussusception. Our patient had a type A dissection where the coronary artery occlusion resulted from compression by the bulging dissected false lumen or by secondary extravasation of blood into the pericardial or perivascular tissue. One unique aspect of this case was the use of a radial approach for the PCI, which may have made it easier to locate the true lumen.

The left main occlusion may represent a slit-like lesion, which can be dynamic due to the compression of the artery, and not necessarily from plaque or thrombus. If these findings are observed, it is important to consider the possibility of dissection. Intravascular ultrasound (IVUS) has been used to help diagnose aortic dissection flap that extends into the coronary artery [6]. In our case, however, the IVUS image is showing the flap outside left main ostium, which has not been described previously.

We found 14 cases of type A aortic dissection presenting as acute myocardial infarction due to left main occlusion in English literature (Table 1) [7-19]. While the definitive treatment is surgery, successful stabilization of the left main artery lesion while awaiting surgery has been reported [8, 12, 15-17, 19]. As PCI techniques have evolved, the management approach has changed from direct surgical approach to PCI of left main artery as the bridge to the definitive surgery. One case even reported the use of PCI and an endograft as a means of repair [8].

**Table 1:** Cases of Type A Aortic Dissection Presenting as Acute Myocardial Infarction Due to Left Main Occlusion

Author	Age	Sex	Presentation	PCI	Surgery	Dx before PCI	Outcome
Tominaga et al, 1999 [7]	52	W	Syncope/shock	NO	Surgery		Discharge
Tominaga et al, 1999 [7]	60	W	STEMI	NO	Surgery		Discharge
Barabas et al, 2000 [8]	74	M	V.fib/STEMI	PCI of LM	Surgery	Yes	Discharge
Pinney and Wasserman, 2002 [9]	46	M	STEMI	NO	Surgery		Discharge
Ohara et al, 2003 [10]	67	M	STEMI/shock	PCI of LM	Not a candidate	No	Discharge/death 5 m later
Cardozo et al, 2004 [11]	68	M	STEMI	PCI of LM	Not a candidate	Yes	Death
Imoto et al, 2005 [12]	71	M	Dissection/shock	PCI of LM	Stent graft	Yes	Discharge
Zegers et al, 2007 [13]	54	M	STEMI/shock	NO	Surgery		Death
Omar et al, 2007 [14]	30	W	STEMI/shock	NO	Surgery		Death
Camaro et al, 2009 [15]	52	M	NSTEMI/shock	PCI of LM	Surgery	No	Discharge
Saxena et al, 2011 [16]	56	M	STEMI	PCI of LM	Surgery		Discharge
Laine et al, 2011 [17]	75	M	STEMI	PCI of LM	Surgery	No	Death
Ravandi and Penny, 2011 [18]	86	M	STEMI	PCI of LM	Not a candidate	Yes	?
Lentini et al, 2013 [19]	70	M	STEMI	PCI of LM	Surgery	Yes	Discharge
Fallahi, 2013	61	M	STEMI	PCI of LM	Surgery	No	Discharge

Early diagnosis is critical in successful treatment of this condition. In our cohort of studies half of the aortic dissections were diagnosed before PCI, and PCI was performed with the intention to stabilize the patient. As illustrated in our case, patients with hemodynamic instability or persistent symptoms may benefit from initial coronary stenting as a bridge to definitive treatment with cardiac surgery.

## References

[1] Spittell PC, Spittell JA, Jr., Joyce JW, Tajik AJ, Edwards WD, Schaff HV, Stanson AW (1993). Clinical features and differential diagnosis of aortic dissection: experience with 236 cases (1980 through 1990). Mayo Clin Proc.

[2] Weber M, Kerber S, Rahmel A, Breithardt G, Diallo S, Bocker W (1997). [Acute thoracic aortic dissection with occlusion of the left coronary artery]. Herz.

[3] Trimarchi S, Nienaber CA, Rampoldi V, Myrmel T, Suzuki T, Mehta RH, Bossone E (2005). Contemporary results of surgery in acute type A aortic dissection: The International Registry of Acute Aortic Dissection experience. J Thorac Cardiovasc Surg.

[4] Kawahito K, Adachi H, Murata S, Yamaguchi A, Ino T (2003). Coronary malperfusion due to type A aortic dissection: mechanism and surgical management. Ann Thorac Surg.

[5] Neri E, Toscano T, Papalia U, Frati G, Massetti M, Capannini G, Tucci E (2001). Proximal aortic dissection with coronary malperfusion: presentation, management, and outcome. J Thorac Cardiovasc Surg.

[6] Na SH, Youn TJ, Cho YS, Lim C, Chung WY, Chae IH, Choi DJ (2006). Images in cardiovascular medicine. Acute myocardial infarction caused by extension of a proximal aortic dissection flap into the right coronary artery: an intracoronary ultrasound image. Circulation.

[7] Tominaga R, Tomita Y, Toshima Y, Nishimura Y, Kurisu K, Morita S, Masuda M (1999). Acute type A aortic dissection involving the left main trunk of the coronary artery--a report of two successful cases. Jpn Circ J.

[8] Barabas M, Gosselin G, Crepeau J, Petitclerc R, Cartier R, Theroux P (2000). Left main stenting-as a bridge to surgery-for acute type A aortic dissection and anterior myocardial infarction. Catheter Cardiovasc Interv.

[9] Pinney SP, Wasserman HS (2002). Anterior myocardial infarction, acute aortic dissection, and anomalous coronary artery. J Interv Cardiol.

[10] Ohara Y, Hiasa Y, Hosokawa S (2003). Successful treatment in a case of acute aortic dissection complicated with acute myocardial infarction due to occlusion of the left main coronary artery. J Invasive Cardiol.

[11] Cardozo C, Riadh R, Mazen M (2004). Acute myocardial infarction due to left main compression aortic dissection treated by direct stenting. J Invasive Cardiol.

[12] Imoto K, Uchida K, Suzuki S, Isoda S, Karube N, Kimura K (2005). Stenting of a left main coronary artery dissection and stent-graft implantation for acute type a aortic dissection. J Endovasc Ther.

[13] Zegers ES, Gehlmann HR, Verheugt FW (2007). Acute myocardial infarction due to an acute type A aortic dissection involving the left main coronary artery. Neth Heart J.

[14] Omar AR, Goh WP, Lim YT (2007). Peripartum acute anterior ST segment elevation myocardial infarction: an uncommon presentation of acute aortic dissection. Ann Acad Med Singapore.

[15] Camaro C, Wouters NT, Gin MT, Bosker HA (2009). Acute myocardial infarction with cardiogenic shock in a patient with acute aortic dissection. Am J Emerg Med.

[16] Saxena P, Boyle A, Shetty S, Edwards M (2011). Left main coronary artery stenting prior to surgical repair of a type a aortic dissection. J Card Surg.

[17] Laine M, Grisoli D, Bonello L (2011). An atypical case of acute myocardial infarction. J Invasive Cardiol.

[18] Ravandi A, Penny WF (2011). Percutaneous intervention of an acute left main coronary occlusion due to dissection of the aortic root. JACC Cardiovasc Interv.

[19] Lentini S, Specchia L, Cricco A, Mangia F, Ignone G, Palmisano D, Di Eusanio G (2013). Hybrid management of acute type A aortic dissection presenting as acute coronary syndrome. Int J Cardiol.

